# Restoration of skilled locomotion by sprouting corticospinal axons induced by co-deletion of PTEN and SOCS3

**DOI:** 10.1038/ncomms9074

**Published:** 2015-11-24

**Authors:** Duo Jin, Yuanyuan Liu, Fang Sun, Xuhua Wang, Xuefeng Liu, Zhigang He

**Affiliations:** 1 F.M. Kirby Neurobiology Center, Children's Hospital and Department of Neurology, Harvard Medical School, 300 Longwood Avenue, Boston, Massachusetts 02115, USA

## Abstract

The limited rewiring of the corticospinal tract (CST) only partially compensates the lost functions after stroke, brain trauma and spinal cord injury. Therefore it is important to develop new therapies to enhance the compensatory circuitry mediated by spared CST axons. Here by using a unilateral pyramidotomy model, we find that deletion of cortical suppressor of cytokine signaling 3 (SOCS3), a negative regulator of cytokine-activated pathway, promotes sprouting of uninjured CST axons to the denervated spinal cord. A likely trigger of such sprouting is ciliary neurotrophic factor (CNTF) expressed in local spinal neurons. Such sprouting can be further enhanced by deletion of phosphatase and tensin homolog (PTEN), a mechanistic target of rapamycin (mTOR) negative regulator, resulting in significant recovery of skilled locomotion. Ablation of the corticospinal neurons with sprouting axons abolishes the improved behavioural performance. Furthermore, by optogenetics-based specific CST stimulation, we show a direct limb motor control by sprouting CST axons, providing direct evidence for the reformation of a functional circuit.

## Abstract (web summary)

A key pathological alteration after brain and spinal cord injury is the disruption of the corticospinal tract (CST) axons that control the voluntary movements. Here the authors show that activating the intrinsic regenerative ability by inhibiting PTEN and SOCS3 expression promotes robust sprouting growth and recovery of skilled locomotion after injury.

## Graphical abstract

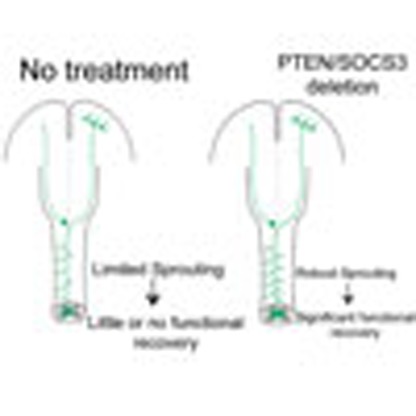

Spontaneous sprouting of spared axons may innervate denervated targets and contribute to naturally occurring functional recovery after injury. However this appears to be very limited in the adult central nervous system (CNS)^1^,^2^,^3^,^4^,^5^. A number of studies have attempted to explore the underlying mechanisms and several manipulations have also been developed to enhance such collateral sprouting^1^,^2^,^3^,^4^,^5^,^6^,^7^,^8^,^9^,^10^,^11^,^12^. For example, both neutralizing extrinsic inhibitory activities and task-specific rehabilitation training are able to promote functional recovery by increasing axonal sprouting in different injury models^5^,^6^,^7^,^8^,^9^,^10^. However, how the sprouting response is initiated after injury remains poorly understood. On the other hand, observed motor function recovery after these treatments are still at most partial. Very often, only the trained motor tasks, but not other motor behaviours, are improved with such manipulations^6^,^10^,^12^,^13^. At some cases, training one task can even have detrimental effects on another task^6^,^10^,^12^. A possible explanation is that rehabilitation training might enhance the functional performance of task-specific circuits at the expense of reducing spared axons available for other behaviours. Therefore, available sprouting axons might be a likely limiting factor in mediating functional recovery.

To develop new strategies of boosting robust sprouting of corticospinal tract (CST) axons, we here attempted to explore how spared (intact) axons sense injury and respond with extensive sprouting. In this study, we show that ciliary neurotrophic factor (CNTF) and perhaps other cytokines, are induced in denervated neurons, might serve as molecular triggers of CST sprouting. By manipulating CNTF and other pathways, we achieved robust CST sprouting, forming new circuits almost as strong as intact ones and significant behavioural functional recovery. Furthermore, by both optogenetic and behavioural approaches, we demonstrated that sprouting CST axons could relay the cortical signals to the spinal cord, controlling the limb movement.

## Results

### SOCS3 deletion in cortical neurons promotes CST sprouting

**Figure 1:**
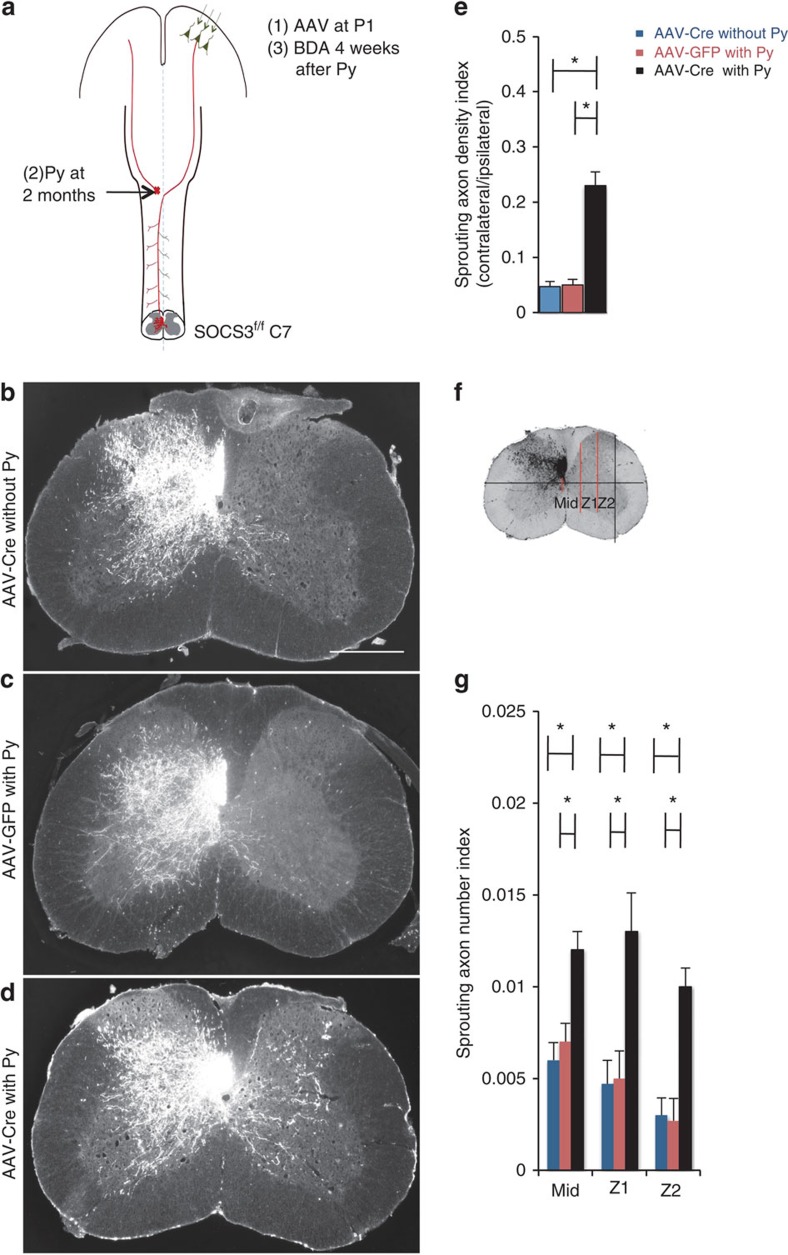
SOCS3 deletion in neonatal cortical neurons increases CST sprouting after unilateral pyramidotomy. (**a**) Scheme of the experiments. AAVs were injected into the sensorimotor cortex of P1–2 SOCS3^f/f^ mice, which then received a unilateral pyramidotomy (Py) or sham lesion at 8 weeks. BDA was injected into the contralateral sensorimotor cortex at 4 weeks post injury and the mice were terminated 2 weeks later. (**b**–**d**) Representative images (*n*=3) of cervical 7 (C7) spinal cord transverse sections from SOCS3^f/f^ mice with cortical AAV-Cre injection and a sham injury (**b**) or with cortical AAV-GFP injection and a unilateral pyramidotomy (**c**) or cortical AAV-Cre injection and a unilateral lesion (**d**). (**e**) Quantification of sprouting axon density index (contralateral/ipsilateral). *P<0.01, ANOVA followed by Bonferroni’s *post hoc* test. (**f**) Scheme of quantifying crossing axons at different regions of the spinal cord (Mid: midline, Z1 or Z2: different lateral positions). (**g**) Quantification of crossing axons counted in different regions of spinal cord normalized against the numbers of labelled CST axons. *P<0.01, ANOVA followed by Bonferroni’s *post hoc* test. For **e**,**g** five mice used in each group. Three sections at the C7 level were quantified per mouse. Scale bar, 500 μm.

Our previous studies showed that deleting SOCS3 in retinal ganglion neurons promotes regeneration of injured optic nerve axons after injury^14^,^15^. We thus asked whether SOCS3 deletion could affect the sprouting of CST axons after unilateral pyramidotomy (Fig. 1a). In this injury paradigm, CST axons from a unilateral cortical hemisphere are transected at the medullary pyramid above the pyramidal decussation. To monitor collateral sprouting from uninjured CST axons, biotinylated dextran amines (BDAs) are injected into the intact side of sensorimotor cortex at indicated post-injury time points, and transverse sections from different levels of the spinal cord are analysed after an additional 2 weeks (Fig. 1a). In control mice, most of the labelled axons are on the contralateral side of the spinal cord, with minimal labelling on the ipsilateral side^7^,^16^,^17^,^18^. Thus, the number of labelled axons on the denervated side of the spinal cord originating from the intact CST in the contralateral side can be used to quantify the extent of CST compensatory sprouting.

To delete SOCS3 in cortical neurons, we first injected Cre-expressing adeno-associated virus (AAV2-Cre) into the intact side of the sensorimotor cortex (contralateral to the injury) of homozygous conditional SOCS3 mutants (SOCS3^f/f^, ref. 19) on postnatal day 1 (P1). This approach has been previously shown to induce efficient Cre-dependent recombination in neurons throughout the sensorimotor cortex^18^. Deletion of SOCS3 at this stage did not appear to change the pattern of CST projections in the adult (Fig. 1b). A unilateral pyramidotomy was performed at 8 weeks of age, and sprouting responses were analysed 6 weeks post injury. SOCS3 deletion significantly increased sprouting from the spared (intact, left) half of the CST into the denervated (right) side of the spinal cord (Fig. 1d,e), comparing to the controls (Fig. 1c,e). In the denervated (right) side of the spinal cord, the labelled axons could be seen in different regions of the grey matter, with the most abundant projections in the intermediate and dorsal spinal cord (Fig. 1d–g). The density of compensatory/collateral sprouting axons amounted to 25% of the uncrossing CST (Fig. 1e). These results suggest that the signalling pathway(s) regulated by SOCS3 regulate the capacity for compensatory sprouting of spared CST axons.

### Spinal CNTF upregulation after unilateral pyramidotomy

SOCS3 is a negative regulator of the Janus kinase/signal transducer and activator of transcription (JAK/STAT) pathway, which is often activated by cytokines such as CNTF^20^. Enhanced CST sprouting from intact cortical neurons after SOCS3 deletion suggests that axonal sprouting responses might be regulated by access to extrinsic cytokines which activate the SOCS3-regulated JAK/STAT pathway. Because the pyramidotomy is performed on one side of the medullary pyramid, we examined the expression of CNTF in the cortex (where CST axons originate) and in the spinal cord (where CST axons terminate) in both intact and injured wild-type mice.

**Figure 2:**
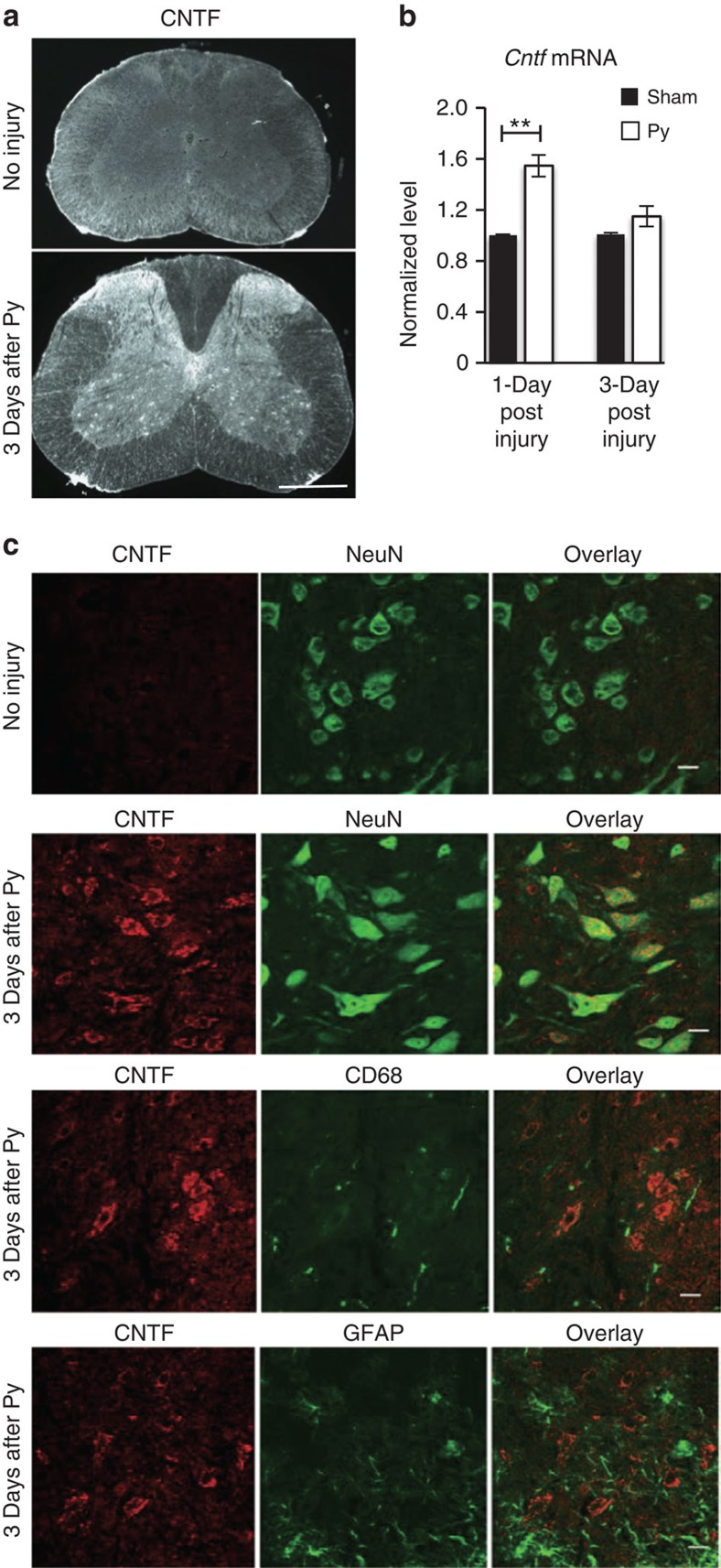
CNTF upregulated expression in denervated spinal cord neurons. (**a**) Representative images (*n*=3) of spinal cord sections stained with anti-CNTF antibodies showing the spinal cord from the mice at 3 days post injury (lower panel), but not intact controls (upper panel) with higher immunoreactivity. Scale bar, 500 μm. (**b**) *Cntf* mRNA measurement by quantitative PCR with reverse transcription of the spinal cord at 1 and 3 days post injury, respectively. *Cntf* mRNA expression levels were normalized to *Gapdh* mRNA levels. **Student’s *t* test, *P*<0.01 samples were from five animals, three replicates. (**c**) Representative images (*n*=3) from the spinal cord of intact (top) or 3 days post injury (the lower three panels) showing the co-staining of anti-CNTF with anti-NeuN (the second panel), but not with anti-CD68 (the third panel) or anti-GFAP (the fourth panel). Scale bars, 20 μm.

As shown in Supplementary Fig. 1, no immunoreactivity with anti-CNTF antibodies could be detected in the cortex after a pyramidotomy on 3 days post injury. However, CNTF immunoreactivity is significantly increased in the spinal cord of the mice with pyramidotomy, in contrast to the low level seen in the spinal cord of control mice (Fig. 2a). Interestingly, similar levels of the CNTF immunoreactivity were seen in both sides of the spinal cord after such unilateral lesion (Fig. 2a). Additionally, by quantitative PCR with reverse transcription to measure the *Cntf* mRNA expression in the cervical spinal cord (C4–C7) after pyramidotomy, we found that compared with the sham control, the CNTF mRNA levels were significantly increased at 1-day post injury (Fig. 2b), suggesting that the CNTF upregulation is likely due to the increased transcription within the denervated spinal cord. Together, these results suggest that a unilateral CST lesion triggers the upregulation of CNTF in the spinal cord.

It is known that glial and inflammatory cells express cytokines such as CNTF after CNS damage such as spinal cord injury^21^,^22^. Thus the observed CNTF induction might be due to a local inflammation as a result of Wallerian degeneration of lesioned CST axons after unilateral pyramidotomy. To test this, we assessed the cell types that express CNTF. By immunohistochemistry, CNTF immnuoreactivity was not detected in CD68+ microglia or GFAP+ astrocytes (Fig. 2c). Instead, most of the CNTF signal was co-localized with NeuN-positive neurons (Fig. 2c), arguing against the possibility that CNTF induction is the direct consequence of glial responses after unilateral pyramidotomy. Furthermore, the CNTF signal was seen in both sides of the spinal cord (Fig. 2a), in contrast to the mainly contralateral projections of CST axons. Therefore, we propose that CNTF induction is likely to occur mainly in denervated spinal neurons, both direct and indirect synaptic targets of CST axons.

### CNTF triggers CST sprouting in intact spinal cord

**Figure 3:**
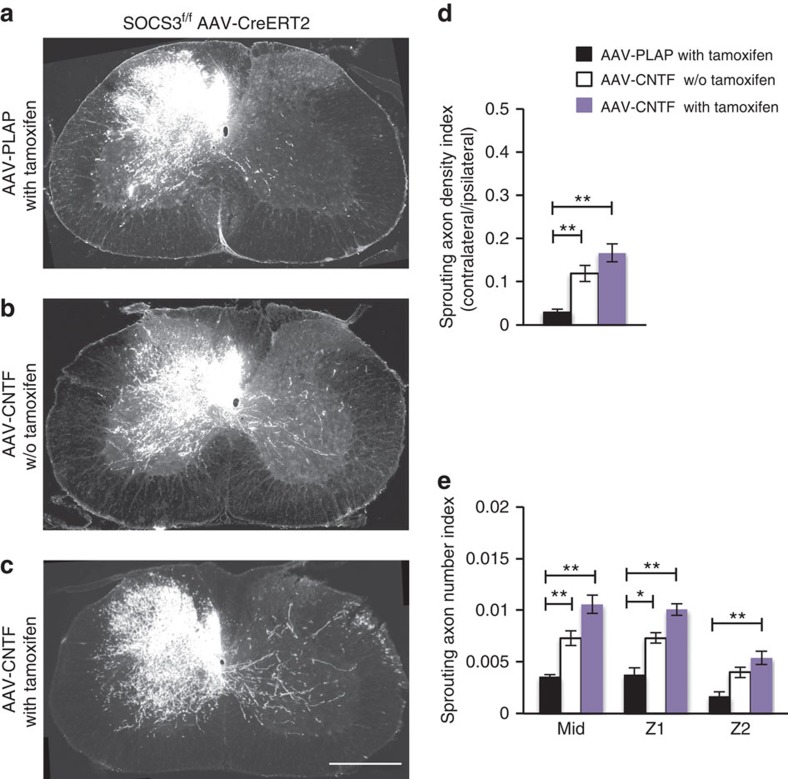
CNTF induces CST sprouting in intact spinal cord. (**a**–**c**) Representative images (*n*=3) from the SOCS3^f/f^ mice with neonatal cortical injection of AAV1-CreERT2 and tamoxifen (**a**,**c**) or oil (**b**) i.p. injection at the age of 6 weeks, intraspinal injection of AAV2-PLAP (**a**) or AAV2-CNTF (**b**,**c**) in the adult (∼20 weeks), and unilateral BDA injection at the somatosensory cortex 4 weeks post intraspinal injection. Scale bar, 500 μm. (**d**) Quantification of sprouting axon density index (contralateral/ipsilateral) in different groups. **P<0.01, ANOVA followed by Bonferroni’s *post hoc* test. (**e**) Quantification of crossing axons counted in different regions of spinal cord normalized against the numbers of labelled CST axons. **P<0.01, *P<0.05 (*P*=0.038), ANOVA followed by Bonferroni’s *post hoc* test. For **d**,**e** three mice used in each group. Three sections at the C6–7 levels were quantified per mouse.

To directly test whether CNTF *per se* is able to stimulate CST sprouting, we unilaterally injected AAV2-CNTF into the cervical spinal cord (C5–C7) of SOCS3^f/f^ mice. These mice were cortically injected with AAV1 that expresses tamoxifen-inducible CreERT2 (AAV1-CreERT2) or control placental alkaline phosphatase (PLAP) at neonatal age and administrated with tamoxifen (or oil as control) in adults. This strategy allows a timely controllable expression of Cre to delete the targeted SOCS3 gene. To validate this, we first injected AAV1-CreERT2 into the cortex of a reporter mouse at neonatal age. Tamoxifen administration resulted in Tomato reporter gene expression in cortical neurons, including pyramidal neurons in layer V (Supplementary Fig. 2). Intraspinal CNTF overexpression (Supplementary Fig. 3) significantly promoted the growth of midline-crossing corticospinal axons labelled by BDA, when compared with the control mice that were intraspinally injected with AAV-PLAP (Fig. 3a,b,d,e). SOCS3 deletion further enhanced the sprouting of CST axons, increasing the density of collateral sprouting axons to ∼20% of the uncrossing CST (Fig. 3b–e). These results suggested that CNTF overexpression in the spinal cord is sufficient to trigger CST sprouting.

### CST sprouting induced by SOCS3 deletion is gp130 dependent

Despite our observation of CNTF induction after injury, basal expression of other cytokines such as LIF and interleukin-6 might also contribute to the sprouting phenotypes. Thus, we examined whether CST sprouting induced by SOCS3 deletion in cortical neurons is dependent on gp130, a required receptor components shared by CNTF and other cytokines. In our initial attempts, we injected AAV-Cre into the cortex of the SOCS3^f/f^/gp130^f/f^ mice at neonatal age and performed a unilateral pyramidotomy in the adult mice. In both gp130^f/f^ and gp130^f/f^/SOCS3^f/f^ mice with neonatal cortical AAV-Cre injection, we found that few CST axons could be labelled by BDA tracing (Supplementary Fig. 4), which might be due to the fact that the JAK/STAT pathway is required for neuronal survival during development^23^,^24^.

**Figure 4:**
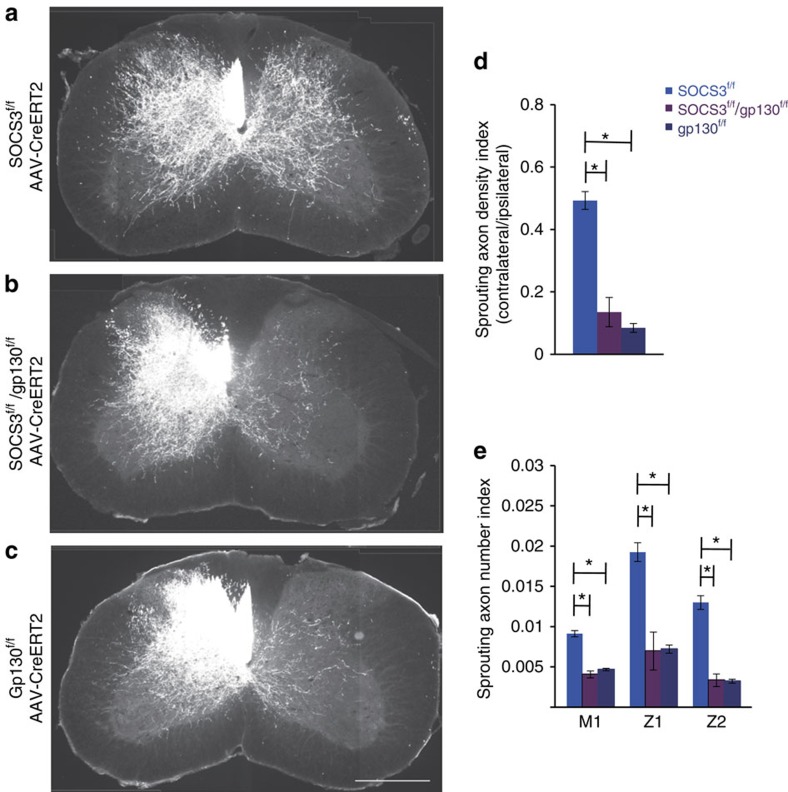
CST sprouting induced by SOCS3 deletion is dependent on gp130. (**a**–**c**) Representative images (*n*=3) from the SOCS3^f/f^ (**a**), gp130^f/f^ (**b**) or SOCS3^f/f^/gp130^f/f^ (**c**) mice with neonatal cortical injection of AAV1-CreERT2 and AAV-ChR-YFP (as a tracer), tamoxifen induction at the age of 6 weeks, and unilateral pyramidotomy at the age of 8 weeks. The mice were sacrificed 12 weeks later. Scale bar, 500 μm. (**d**) Quantification of sprouting axon density index (contralateral/ipsilateral). *P<0.01, ANOVA followed by Bonferroni’s *post hoc* test. (**e**) Quantification of crossing axons counted in different regions of spinal cord normalized against the numbers of labelled CST axons. *P<0.01, ANOVA followed by Bonferroni’s *post hoc* test. For **d**,**e** five mice used in each group. Three sections at the C7 level were quantified per mouse.

We then injected AAV1-CreERT2 and AAV-channelrhodopsin (ChR)-YFP to the cortex of the SOCS3^f/f^, gp130^f/f^ or SOCS3^f/f^/gp130^f/f^ mice at a neonatal age, and performed tamoxifen induction at 6 weeks, followed by unilateral pyramidotomy at 8 weeks. In these experiments, ChR-YFP was utilized as a tracer. In another 3 months, we analysed CST axon sprouting from the intact cortex. As expected, robust CST sprouting was seen in the SOCS3-deleted mice (Fig. 4a,d,e). The observed sprouting was stronger than what seen at 6 weeks after injury (Fig. 1d), suggesting a continuous sprouting response as a result of SOCS3 deletion. However, co-deletion of gp130 and SOCS3 dramatically reduced sprouting (Fig. 4b,d,e). Comparable labelling efficiency of CST by ChR-YFP suggested that neuronal death is unlikely to contribute to different sprouting in these mouse lines (Fig. 4a–c). The residual sprouting seen in mice with gp130 deletion might reflect the contribution of other cytokine-independent mechanisms.

### Enhanced CST sprouting induced by PTEN/SOCS3 deletion

The results above suggest that denervation-triggered neuronal CNTF expression might be an important extrinsic regulator of CST collateral sprouting. We next examined whether increasing intrinsic growth capacity in uninjured CST neurons by PTEN deletion could further increase CST sprouting elicited by SOCS3 deletion. Thus, by following the procedure shown in Fig. 1a, we injected AAV-Cre or AAV-GFP to the sensorimotor cortex of PTEN^f/f^/SOCS3^f/f^ mice at P1, performed a unilateral pyramidotomy at 8 weeks of age, and analysed the extent of CST sprouting at 6 weeks post injury.

**Figure 5:**
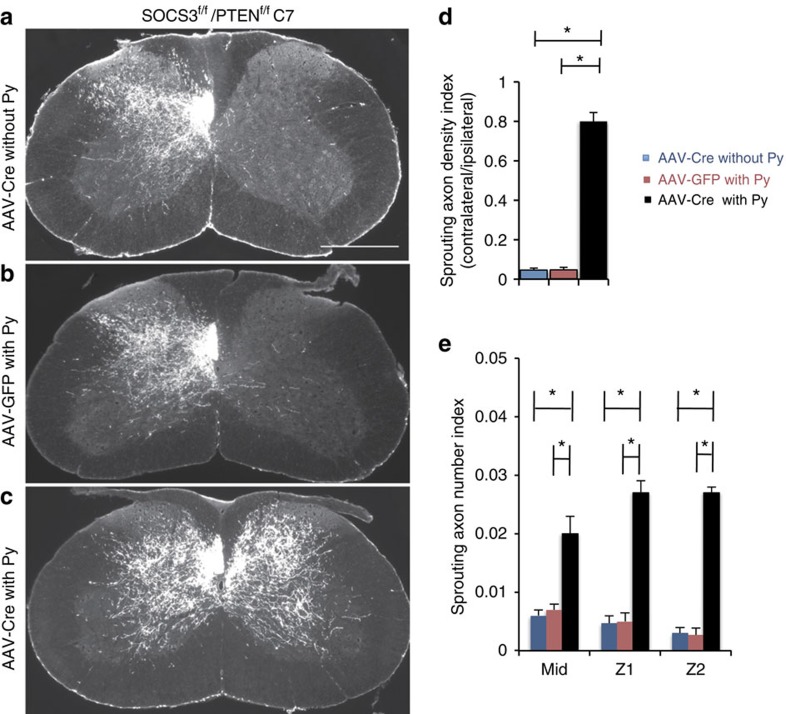
Significant CST sprouting in SOCS3 and PTEN co-deleted mice after unilateral pyramidotomy. (**a**–**c**) Representative images (*n*=3) of cervical 7 (C7) spinal cord transverse sections from SOCS3^f/f^/PTEN^f/f^ mice with cortical AAV-Cre injection and a sham injury (**a**) or with cortical AAV-GFP injection and a unilateral pyramidotomy (Py) (**b**) or cortical AAV-Cre injection and a Py (**c**). Scale bar, 500 μm. (**d**) Quantification of sprouting axon density index (contralateral/ipsilateral). *P<0.01, ANOVA followed by Bonferroni’s *post hoc* correction. (**e**) Quantifications of crossing axons counted in different regions of spinal cord normalized against the numbers of labelled CST axons. *P<0.01, ANOVA followed by Bonferroni’s *post hoc* correction. For **d**,**e** five mice were used in each group. Three sections at the C7 level were quantified per mouse.

In intact PTEN and SOCS3 double-deleted mice, the CST projection pattern remains unaltered (Fig. 5a). However, after unilateral pyramidotomy, uninjured CST axons underwent dramatically increased sprouting responses (Fig. 5c–e), to an extent significantly stronger than that seen in the single SOCS3-deleted (Fig. 1c) or single PTEN-deleted mice^18^. In these double mutants, sprouting axons reached almost the entire CST territory in the denervated side of the spinal cord (Fig. 5c,d), although their overall distribution is similar to that seen in the single mutants (Fig. 5e versus Fig. 1g). Most, if not all, of these labelled CST axons are likely to be the midline-crossing axons from the contralateral side, because little staining was observed in the white matter of the denervated side of the spinal cord (Fig. 5c). These results suggest that co-deletion of PTEN and SOCS3 greatly enhances corticospinal neuron sprouting after a unilateral pyramidotomy.

### Improved skilled locomotion by PTEN/SOCS3 deletion

**Figure 6:**
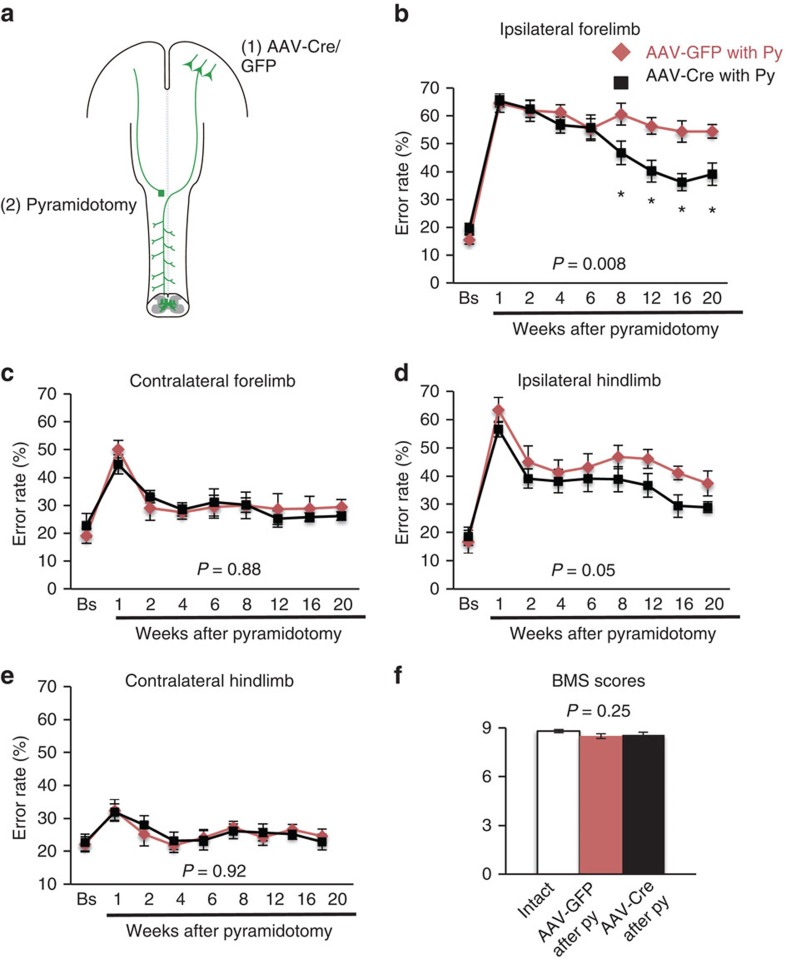
Significant recovery of skilled locomotion in mice with neonatal SOCS3 and PTEN co-deletion after unilateral pyramidotomy. (**a**) Experimental paradigm: mice were injected with AAV-GFP or AAV-Cre at neonatal stage and subjected to unilateral pyramidotomy in adults. (**b**–**e**) Performance on irregularly horizontal ladder of ipsilateral (**b**,**d**) and contralateral (**c**,**e**) forelimbs (**b**,**c**) and hindlimbs (**d**,**e**) to the lesion, respectively. *P* values: repeated-measures ANOVA, *P<0.05 (*P*=0.039, 0.019, 0.005, 0.015 for 8, 12, 16 and 20 week respectively), Bonferroni’s *post hoc* correction (*n*=11 and 12 for AAV-GFP and AAV-Cre injected group, respectively). (**f**) BMS scores of intact mice and the PTEN^f/f^/SOCS3^f/f^ mice with AAV-GFP or AAV-Cre injection at 20 weeks after Py. *P*=0.25, one-way ANOVA (*n*=12, 11 and 12 for intact, PTEN^f/f^/SOCS3^f/f^ mice with AAV-GFP or AAV-Cre injection, respectively).

To assess skilled motor outcomes in these animals, we used a horizontal walking assay in which the animals were trained and tested to walk on a horizontal ladder with irregularly spaced rungs^25^,^26^. Previous studies suggested that CST-carried cortical commands are required for animals to prevent missteps during their walking on such irregularly spaced ladders^25^,^26^,^27^,^28^,^29^. In this double-blinded experiment, AAV-Cre or control AAV-GFP vectors were injected to the cortex of neonatal PTEN^f/f^/SOCS3^f/f^ mice (Fig. 6a). At the age of 8 weeks, the animals were allowed to be familiar with the ladder till they crossed the ladder with a forelimb error ∼20%, before unilateral pyramidotomy (Fig. 6b,c).

After injury, the animals in both groups showed similar degrees of deficit, but the differences started to become statistically significant ∼8 weeks post injury on the injured forelimbs (Fig. 6b), but not hindlimbs (Fig. 6d). Temporally delayed functional recovery might reflect the need of spontaneous synaptic formation and refinement of sprouting axons for their functional integration. Such improvement further increased over 8–16 weeks, but appeared to reach a plateau around the time of 16–20 weeks post injury (Fig. 6b). As a control, the error rates of the uninjured side of both forelimbs and hindlimbs were comparable in these two groups over the entire course of the experiments (Fig. 6c,e).

On the other hand, we scored the unskilled locomotion of the mice in different groups at 20 weeks post injury by Basso Mouse Scale (BMS)^30^ (Fig. 6f). No significant difference was seen between intact mice and those with unilateral pyramidotomy, consistent with the notion that CST axons have little influence on unskilled locomotor function^31^. We also failed to see significant difference between the PTEN^f/f^/SOCS3^f/f^ mice with cortical injection of AAV-GFP or AAV-Cre (Fig. 6f). Together, these results suggested that the PTEN and SOCS3 deletion in cortical neurons allow the CST-deprived forelimb to restore skilled locomotor function.

### Limb movement induced by optogenetic CST stimulation

Because cortical neurons can control the spinal cord function by direct CST or indirect relay pathways, we next utilized an optogenetic approach to specifically stimulate CST axons to address whether the observed behavioural improvement results from the sprouting CST axons-mediated regained control of the denervated spinal cord.

**Figure 7:**
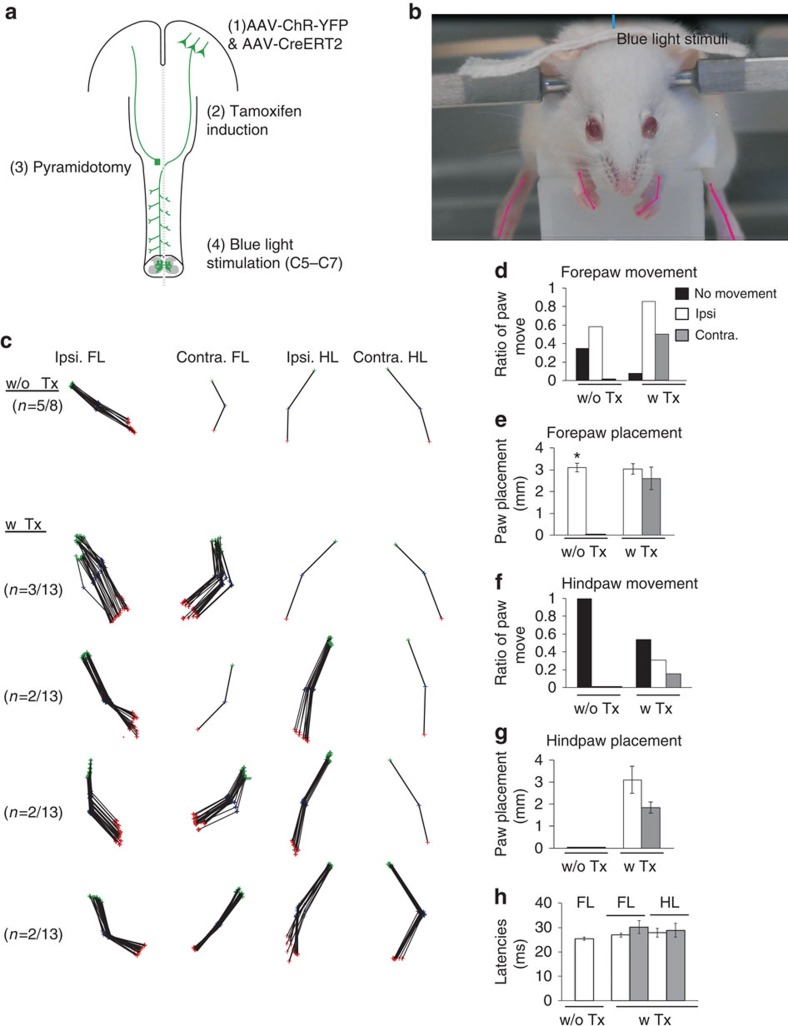
CST-specific optogenetic stimulation induces limb movement. (**a**) Experimental paradigm of optogenetic stimulation. PTEN^f/f^/SOCS3^f/f^ mice received cortical injection of AAV1-ChR-YFP and AAV-CreERT2 at P1 (1), tamoxifen at 6 weeks (2), unilateral pyramidotomy at 8 weeks (3), and subjected to optogenetic stimulation at C5–C7 levels ∼12 weeks after injury (4). (**b**) Illustration of the optogenetic stimulation apparatus. White arrow indicates blue light stimuli. Red dots with connected lines delineate specific joints for trajectory analysis. (**c**) Representative trajectories (*n*=8 for control group, *n*=13 for experimental group) of paw movements induced by photostimulation in control and PTEN/SOCS3-deleted mice. In contrast to the control group (five out of eight animals showed only ipsilateral forelimb movement), the mice with PTEN/SOCS3 co-deletion showed different paw movement patterns, on CST specific, optogenetic stimulation. These include bilateral forelimb movement (3/13), ipsilateral forelimb and hindlimb movement (2/13), bilateral forelimb movement and ispilateral hindlimb movement (2/13) and bilateral forelimb and hindlimb movement (2/13). Representative movies are shown in Supplementary Material. (**d**–**g**) Quantification of paw movement patterns (**d**,**f**), paw placement (**e**,**g**), and latencies of the movement onset (**h**) for specific forepaws and hindpaws on photostimulation in two groups of mice. *P<0.01, Student’s *t* test, NS, not significant, one-way ANOVA. Eight and thirteen animals were used for quantification in animals without or with tamoxifen induction, respectively. Three trajectories were quantified per mouse.

In control mice, a unilateral cortical injection of AAV1 expressing the fusion protein of channelrhodopsin and YFP (AAV-ChR-YFP) led to the YFP expression in the neuronal soma as well as the CST axons in the spinal cord (Fig. 7a, Supplementary Fig. 5a–c). We first characterized the stimulation protocol in which the blue laser was used to stimulate the CST on the dorsal surface of the C5–7 spinal cord in these mice (Fig. 7b, Supplementary Fig. 6a). The amplitude of the movement increased with the longer stimulation duration, peaked at 200 ms and decreased at 2 s or longer (Supplementary Fig. 6a,b). In contrast, we failed to see movement of the contralateral forelimb or hindlimbs in these trials (Supplementary Fig. 6a). Furthermore, after cutting the dorsal column of the spinal cord at C2–3 level, similar light stimulation at the spinal cord rostral to the lesion level failed to trigger the movement of the ipsilateral forelimb (Supplementary Fig. 6a, the bottom panel), arguing against the possibility that the forelimb movement is due to the antidromic transmission of CST and activation of the other indirect descending tracts. Together, our results suggest that the forelimb movement after photostimulation is likely due to orthodromic CST transmission, and we therefore selected 200 ms as the stimulation length for further experiments.

Next, we co-injected AAV-ChR-YFP and AAV-CreERT2 into the one side cortex of the neonatal PTEN^f/f^/SOCS3^f/f^ mice. The animals were treated with or without tamoxifen at the age of 6 weeks and a unilateral pyramidotomy procedure was performed at the age of 8 weeks. Similar to wild-type mice (Supplementary Fig. 6), only the ipsilateral forelimb responded to the stimulation in mice without tamoxifen induction (Fig. 7c–h, Supplementary Movie 1). However, in the SOCS3^f/f^/PTEN^f/f^ mice with tamoxifen induction, most mice showed robust bilateral forelimb movements (Fig. 7c–h, Supplementary Movie 1), often with the flexion of the shoulder and elbow joints (Fig. 7c). The latencies of the limb movements, depending on the laser power and the expression levels of ChR receptors, were indifferent between the ipsilateral and the contralateral forelimbs in the SOCS3/PTEN-deleted mice (Fig. 7h), suggesting that the contralateral forelimb movements were likely mediated by re-wired circuits formed by the collaterally sprouted CST axons. The differences on optogenetic-induced forelimb movements between control and experimental animals were unlikely due to different expression of chR2-YFP in cortical neurons or CST axons in the spinal cord (Supplementary Fig. 7). These results, therefore, suggest that the contralateral forelimb movement in mice with SOCS3/PTEN co-deletion is likely due to CST sprouting-mediated new circuits between the intact CST and the denervated side of the spinal cord.

Interestingly, in some of the injured mice with PTEN/SOCS3 co-deletion, the photostimulation at the C5–7 levels also resulted in the hindlimb movement (Fig. 7c,f,g), which was never seen after cervical CST stimulation in control animals (Fig. 7c top panel). In these PTEN/SOCS3-deleted mice, we found that the CST sprouting not only occurred at the cervical level, but also in the lower spinal cord levels, such as lumbar enlargements (Supplementary Fig. 8). However, not all animals with PTEN and SOCS3 deletion showed light-induced hindlimb movements (Fig. 7c), possibly due to a lesser extent of CST sprouting seen at the lumbar level (Supplementary Fig. 8). It remains to be determined whether hindlimb movements are the direct result of such lumbar CST sprouting or the indirect consequences of forelimb movements induced by cervical CST sprouting through propriospinal axons that connects different segments of spinal cord^17^. Nevertheless, our results provide direct evidence for functional circuits mediated by sprouting CST axons.

### Abolishment of the improved skilled locomotion by selective ablation of corticospinal neurons with sprouted axons

**Figure 8:**
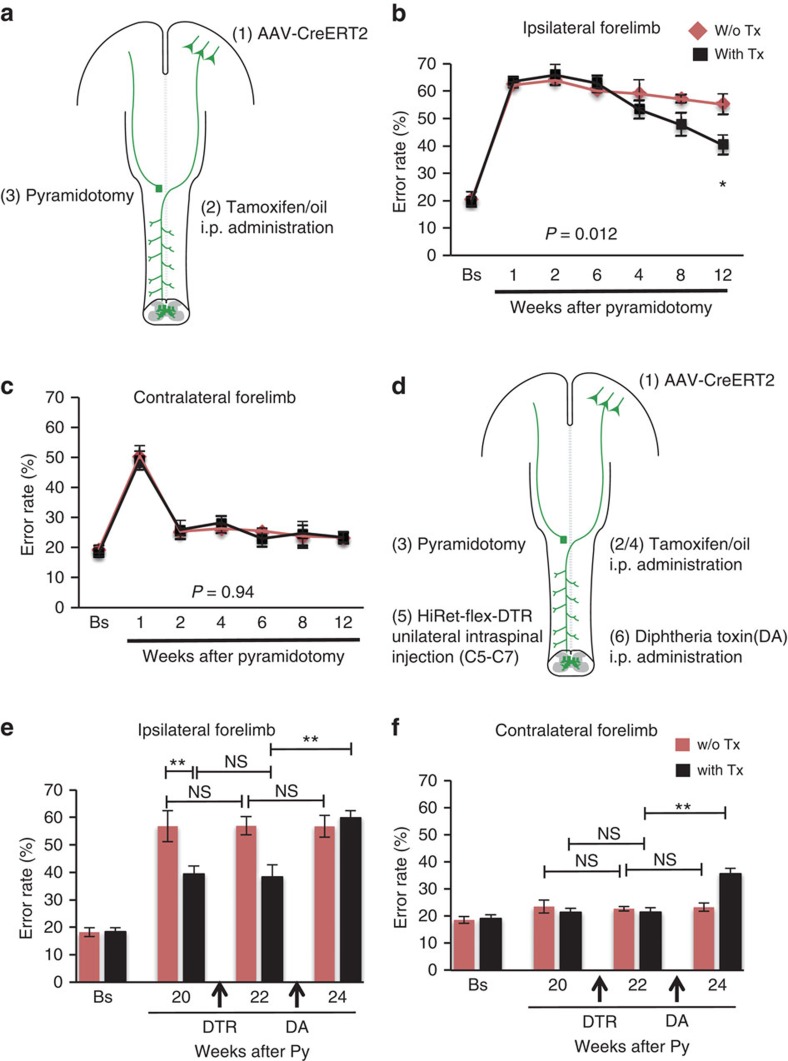
Ablation of cortical neurons with sprouting axons abolishes recovered skilled locomotion performance. (**a**) Experimental paradigm: mice received cortical injection of AAV-CreERT2 at neonatal stage, tamoxifen at the age of 6 weeks, and unilateral pyramidotomy at 8–10 weeks, the same as Fig. 6a. (**b**,**c**) Performance on irregularly horizontal ladder of ipsilateral (**b**) and contralateral (**c**) forelimbs to the lesion, respectively. *P* values: repeated-measures ANOVA, *P<0.05 (*P*=0.03), Bonferroni’s *post hoc* correction (*n*=9 and 10 for without and with tamoxifen injected group, respectively). (**d**) Experimental paradigm: the same group of animals used in **a**–**c** were further analysed here. At 20 weeks after pyramidotomy, tamoxifen was administrated again and lentivirus (HiRet-FLEX-DTR) was intraspinally injected into the denervated side of the cervical spinal cord (C5–C7). After 2 weeks, diphtheria toxin was administrated (i.p.). (**e**,**f**) Performance on irregularly horizontal ladder of ipsilateral (**e**) and contralateral (**f**) forelimbs to the lesion, respectively. **P<0.01, NS, not significant, Student’s *t* test (*n*=6 and 4 for without and with tamoxifen injected group, respectively).

Next, we performed a separate set of experiments with PTEN and SOCS3 co-deletion from the age of 6 weeks and unilateral pyramidotomy (Fig. 8a, the same as Fig. 7a) and the mice were assays for their performances in the irregularly spaced horizontal walking assay (Fig. 8). Similar to what seen in the mice with neonatal PTEN and SOCS3 co-deletion (Fig. 6), the injured forelimb showed significant reduction of error rate (Fig. 8b,c). This behavioural improvement occurred gradually and became statistically significant from 12 weeks post injury (Fig. 8b).

To assess the contribution of sprouting CST axons in the observed functional recovery, we adapted an intersectional targeting strategy to ablate the CST neurons with their axons sprouted into the denervated side of the spinal cord^8^,^32^,^33^,^34^. To do this, we first optimized a procedure of stereotaxic injection into the unilateral spinal cord (Supplementary Fig. 9). With this, we injected the highly efficient retrograde gene transfer lentiviral vector (HiRet) carrying the flip-excision (FLEX)-human diphtheria toxin receptor (DTR) into the denervated side of the cervical spinal cord (C5–C7) at 20 weeks post injury (Fig. 8d). Because these animals were previously injected with AAV-CreERT2 in the ipsilateral cortex (Fig. 8d), only tamoxifen-induced CreERT2-expressing cortical neurons that sprouted midline-crossing axons would express DTR and become susceptible to diphtheria toxin-mediated ablation. We first reassessed animals’ performance on irregularly spaced horizontal ladder and found that the intraspinal virus injection had no overt effect on their skilled limb movement (Fig. 8e). Two weeks after diphtheria toxin administration, the improved performance of denervated forelimbs significantly declined (Fig. 8e), along with a significant reduction of the collaterally sprouted axons in the denervated side (Supplementary Fig. 10). The performance of the uninjured forelimbs was also affected to some extent (Fig. 8f), likely because the ablation of these cortical neurons impacted not only the sprouting axons in the denervated side but also the physiological projections in the intact side of the spinal cord (Supplementary Fig. 10). In contrast, no significant effects were observed in control animals (without tamoxifen-induced Cre expression; Fig. 8d–f). These results suggested that the sprouted axons from intact corticospinal neurons are required for the recovered performance of the precise walking task. Together, our results support the notion that sprouting CST axons induced by PTEN and SOCS3 co-deletion are able to mediate the functional recovery of injured forelimbs.

## Discussion

Collateral sprouting of spared axons has been proposed as a key mechanism for spontaneous functional recovery after CNS injuries. However, its underlying mechanisms remain elusive and its precise contribution to functional restoration has not been definitively addressed. While removing axon growth-inhibitory molecules has been shown to promote CST sprouting^1^,^6^,^7^,^8^, manipulation of intrinsic regenerative ability has emerged as a powerful strategy of enhancing axon regrowth in the adult CNS^14^,^15^,^18^. However, in light of recent finding about neuron type-specific enhancement of axon regeneration by PTEN deletion^35^, it is unknown whether SOCS3-dependent STAT3 pathway is able to regulate CST regrowth. In addition, since manipulating these pathways in adult neurons might alter their cellular and molecular properties, whether these affected neurons could still form functional neuronal connections remain unknown. In this study, by using a unilateral pyramidotomy model, we identified CNTF derived from denervated neurons as an important molecular trigger for CST sprouting. Enhancing this CNTF-dependent STAT3 pathway and co-activating mTOR activity, by co-deletion of SOCS3 and PTEN genes in either neonatal or adult cortical neurons, resulted in robust CST sprouting with an accompanying functional recovery of forelimb skilled movements. Such functional recovery was reversed by selectively ablating corticospinal neurons that collaterally sprouted midline-crossing axons in the spinal cord. By using optogenetics-assisted methods, we were able to achieve specific stimulation of CST axons and demonstrate direct effects on limb movements, suggesting direct control of forelimb movement by sprouting axons.

Previous studies have suggested neurotrophic factors, such as brain-derived neurotrophic factor (BDNF)^36^, and axon guidance molecules such as Slit and Ephrins^37^, as sprouting inducers. Our results here implicate CNTF and perhaps other cytokines as important endogenous triggers for CST sprouting after unilateral corticospinal lesion. gp130 Deletion abolishes most of observed sprouting growth, suggesting that cytokines play a key role in this process. CNTF itself is sufficient to trigger CST sprouting even in the intact mice. In this regard, cytokine-dependent JAK/STAT pathway has been shown to be critical for regenerative growth, but not developmental growth, in cultured sensory neurons^38^. Different from reported expression of cytokines in glial and inflammatory cells^21^,^22^, our results suggest that local spinal neurons might respond to denervation by upregulating CNTF expression, which subsequently stimulate axonal sprouting and possibly functional compensation. Such cytokine upregulation by direct and indirect denervated neurons might suggest a potential molecular mechanism for widely reported post-injury structural and functional reorganizations in the CNS, such as motor cortex reorganization seen after forelimb amputation^39^,^40^.

Furthermore, our results provide direct evidence for the notion that sprouting axons, as a result of manipulating these pathways, are able to form new functional circuits, mediating skilled locomotion functional recovery. In these experiments, the observed partial and delayed functional recovery might reflect inaccurate synaptic connections. In light of recent studies that rehabilitation could promote the functional refinement of sprouting^6^,^8^,^9^,^10^,^11^,^12^, future studies should be aimed at deciphering other accessory treatments that maximize the extent of functional restoration induced by manipulating SOCS3 and mTOR-dependent pathways.

## Methods

### Mice and surgeries

All experimental procedures were performed in compliance with animal protocols approved by the Institutional Animal Care and Use Committee at Children’s Hospital, Boston. For cortical AAV injection, neonatal PTEN^f/f^, SOCS3^f/f^ or PTEN^f/f^/SOCS3^f/f^ mice (both sexes, P1) were anaesthetized and injected with 2 μl of either 10^12^ GC/ml AAV-Cre or AAV-GFP into four sites of the sensorimotor cortex contralateral to the pyramidotomy side using a nanolitre injector attached to a fine glass pipette. Mice were then placed on a warming pad and returned to their mothers after regaining normal colour and activity. For pyramidotomy, mice (both sexes, 8 weeks) were anaesthetized with ketamine/xylazine. The procedure is similar to that described previously^18^. Briefly, an incision was made at the one side of the trachea. Blunt dissection was performed to expose the skull base and a craniotomy allowed for access to the medullary pyramids. The pyramid was cut with a fine scalpel medially up to the basilar artery. BDA tracing was performed 4 weeks later (see below). For cervical spinal injection of AAVs expressing PLAP/CNTF (both sexes, 12–14 weeks) or HiRet-flex-DTR (both sexes, 30–32 weeks), we followed the procedure described elsewhere^8^. Briefly, mice were anaesthetized with ketamine/xylazine. A minimally invasive laminectomy was made at the cervical level C5–C7. Virus was loaded into a pulled glass capillary attached to the nanolitre. The nanolitre was then mounted to the stereotaxic frame and the glass capillary was inserted into the cervical spinal cord. Virus was injected at 3 points (100 nl per point) through ventral to dorsal spinal cord per injection tract. Three injection tracts (from middle to lateral) were made on individual side of the spinal cord. For unilateral spinal cord injection (both sexes, 12 weeks), CTB was co-injected with virus to monitor the injection locations.

### BDA tracing

To label CST axons by anterograde tracing, we injected a total of 2.0 μl of BDA (10%, Invitrogen, D-1956) into the right sensorimotor cortex at five sites (anterior–posterior coordinates from Bregma in mm: 1.0, 0.5, 0, −0.5, −1.0, all at 1.0 mm lateral and at a depth of 0.5 mm). Mice were kept for an additional 2 weeks before being sacrificed.

### Histology and immunohistochemistry

Mice were given a lethal dose of anaesthesia and transcardially perfused with 4% paraformaldehyde. Brains and spinal cords were isolated and post-fixed in the same fixative overnight at 4 °C. Tissues were cryoprotected via increasing concentrations of sucrose. After embedding into OCT compound, the samples were snap frozen in dry ice. Serial sections (25 μm) were collected and stored at −20 °C until processed. Coronal sections of the lower medulla were cut for counting BDA-labelled CST fibres. For assessing the extent of CST sprouting, serial sections at C7 and other levels of the spinal cords were cut in the transverse plane. Immunostaining was performed following standard protocols. All antibodies were diluted in a solution consisting of 5% normal goat or donkey serum and 1% Triton X-100 in phosphate-buffered saline (PBS). We used goat antibody to CNTF^41^ (5 μg ml^−1^, R&D Systems, AF-557-NA), rat antibody to CD68 (ref. 42; 1:200, Serotec, MCA1957), rabbit antibody to GFAP^43^ (1:200, Wako, Z0334) and mouse antibody to NeuN (1:100, Millipore, MAB377). Sections were incubated with primary antibodies overnight at 4 °C and washed three times for 10 min with PBS. Secondary antibodies (biotin-conjugated donkey antibody to goat and Alexa 488–conjugated goat antibody to rabbit, rat and mouse) were then applied and incubated for 1 h at 20–25 °C. For CNTF staining, Elite Avidin biotin Conjugate (ABC, Vector Lab) was applied, followed by TSA Cyanine 3 (Perkin Elmer). To detect BDA-labelled fibres, BDA staining was performed by incubating the sections in PBS containing streptavidin–horseradish peroxidase. The remaining staining procedure was performed according to the protocol provided by TSA Cyanine 3 system (Perkin Elmer).

### mRNA quantification

Adult mice with pyramidotomy or sham surgery were euthanized at 1 day or 3 days (*n*=3 for each condition) post surgery. Tissues from the cervical spinal cord (C4–C7) were dissected out immediately in cold 1 × PBS. After homogenization, total RNAs were extracted (Trizol, Invitrogen), reversely transcribed (Superscript III, Invitrogen) and subject to quantitative PCR (CFX Connect, BioRad) to measure *Cntf* mRNA expression levels in the denervated spinal cord. *Gapdh* was used for loading control. The primers used for *Cntf* & *Gapdh* were: forward:5′-GGTGACTTCCATCAGGCAATA; reverse: 5′-GCATCCCATCAGCCTCTTT; forward: 5′-AACAGCAACTCCCACTCTTC; reverse: 5′-CCTGTTGCTGTAGCCGTATT, respectively.

### Axon number and fluorescence intensity quantification

Digital images of C5–7 levels of the spinal cord transverse sections were collected using a Nikon fluorescence microscope. Densitometry measurement on each side of the grey matter was taken using Metamorph software, after being subthresholded to the background and normalized by area. The outcome measure of the sprouting density index was the ratio of contralateral and ipsilateral counts. To quantify the number of sprouting axons, we followed the methods used in our previous studies^16^. Briefly, we drew a horizontal line through the central canal and across the lateral rim of the grey matter. Three vertical lines were then drawn to divide the horizontal line into three equal parts, starting from the central canal to the lateral rim. Only axons crossing the three lines were counted in each section. The results were presented after normalization with the number of counted CST axons at the medulla level. At least three sections were counted for each mouse. To compare the expression of chR2-YFP in the cortex layer V and in the CST axons in the spinal cord, we measured the averaged fluorescent intensities in transverse sections from the animals (three for each group) that were used for the optogenetic stimulation experiment. For layer V cortex, five sections that cover the chR2-YFP expressed areas were selected per animal. For CST axons in the spinal cord, three sections from the cervical spinal cord C5–C7 were selected per animal. ImageJ was used to calculate the mean fluorescent intensities.

### Photostimulation of CST axons with kinematic analysis

A mixture of AAV1-ChR-YFP and AAV1-CreERT2 was injected into the motor and somatosensory cortex of mice at the age of P1. The mice were divided into two groups. The experimental group received tamoxifen induction at 6 weeks while the control group was injected with corn oil. The pyramidotomy was performed at 8 weeks. Twelve weeks later both groups of mice were anaesthetized with ketamine and xylazine and the cervical segments (C3–C7) of the spinal cord were exposed. To ensure the constant, same level of anaesthesia between different animals, we closely monitored animals’ breathing rate (∼100 per min). The maintenance dose of ketamine/xylazine was administrated approximately every 30 min or when the breathing rate dramatically changed^44^. The mice were fixed in the stereotaxic frame with the four limbs hanging in the air for the video recording. Light was delivered using an optic fiber connected to the ferrule via a sleeve. A 473 nm diode laser (Crystalaser) and an arbitrary waveform generator were used to deliver the pulse of light (3, 5, 10, 20, 100, 200 ms and 5 s).

Videos were analysed by Simi Motion software (Simi Motion 7.5, Simi Reality Motion Systems GmbH). We considered responses as optogenetically evoked only if the latencies were longer than 17 ms and the amplitudes were >2 s.d. above the mean amplitude of all movements within 500 ms before stimulation onset. The anatomical landmarks for joints for bilateral forepaws (digit tip, Metacarpophalangeal joint, wrist) and hindpaws (digit tip, metatarsophalangeal joint, ankle) were identified and manually traced for kinematic analysis. Paw placement was calculated as the vertical distance for each paw movement.

### Horizontal ladder walking and BMS scoring

For horizontal ladder walking assay, mice in different groups were tested to walk on a horizontal ladder with irregular spacing between rungs, following the procedure described previously^25^,^26^,^27^. Briefly, the ladder was elevated 30 cm above the ground. Animals were trained to cross the ladder until their performance achieved the plateau (with a forelimb error ∼20%). To prevent animals from learning the pattern, the irregular pattern was changed from trial to trial. All trials were video recorded (Hotshot e64) and paw placement was analysed twice by blinded observers. We define steps with placement of the palm of the paw on the rung and digits closed as correct contact. All other steps were recorded as errors, and the results were expressed as percentage errors. Baseline error rates were established with at least two testing sessions within the 5 days before lesion. Testing was performed 5 days before the pyramidotomy and at 1, 2, 4, 6, 8, 12, 16 and 20 weeks after injury without additional training between each time point. For BMS test, animals with AAV-GFP or AAV-Cre injection were blindly assessed for BMS scores at 20 weeks after pyramidotomy by two independent investigators, following the method^30^. Intact animals were scored in parallel as controls.

### Selective ablation of corticospinal neurons with sprouted axons to the denervated side of the spinal cord

Mice received cortical injection of AAV-CreERT2 at neonatal stage, tamoxifen (or oil as control) at the age of 6 weeks, and unilateral pyramidotomy at 8–10 weeks. Twenty weeks after injury, tamoxifen (or oil) was injected (i.p.) again to ensure the activation of CreERT2. A laminectomy was performed at C5–7 level. The viruses (1.2 × 10^12^ copies per ml) generated by a HiRet^32^ carrying the FLEX-DTR were stereotaxically injected into the denervated side of the spinal cord of the tamoxifen or oil injected animals. After 2 weeks, animals were tested on the irregularly spaced horizontal ladder to reassess their performance of the skilled limb movement. Diphtheria toxin was then administrated (100 μg kg^−1^, i.p.). Animals were tested on the horizontal ladder again 2 weeks after diphtheria toxin administration.

### Statistical analysis

The normality and variance similarity were measured by STATA (version 12, College station, TX, USA) before we applied any parametric tests. Error bars in all figures represent mean±s.e.m. For Figs 1, 3, 4 and and Supplementary Fig. 10, five and three mice were used in each group, respectively. Three sections at the C6–7 levels were quantified per mouse. One-way ANOVA was performed and Bonferroni’s *post hoc* correction was applied for comparison between individual groups. For Figs 2 and 7, and Supplementary Figs 7 and 8, unpaired Student’s *t* test (*n*=5 and 5) was applied. For Figs 2 and 3 blots were used for quantification. Each blot is from the homogenised tissue from four animals. For Fig. 6 (*n*=11 and 12 for AAV-GFP and AAV-Cre injected group, respectively), Fig. 8b,c (*n*=9 and 10 for without and with tamoxifen injection group, respectively), repeated-measures ANOVA was used, followed by Bonferroni’s *post hoc* correction for the comparisons of individual groups. For Fig. 6f, one-way ANOVA (*n*=12, 11 and 12) was applied. For Figs 7 and 8 (without tamoxifen induction) and 13 (with tamoxifen induction) animals were included, three trajectories per animal were used for quantification. For Fig. 8e,f, unpaired Student’s *t* test (*n*=6,4) was applied. *Post hoc* power analysis, which achieved a power of 91.67% (Fig. 6) and 88.23% (Fig. 8), respectively, was used to demonstrate a sufficient sample size.

## Additional information

**How to cite this article:** Jin, D. *et al.* Restoration of skilled locomotion by sprouting corticospinal axons induced by co-deletion of PTEN and SOCS3. *Nat. Commun.* 6:8074 doi: 10.1038/ncomms9074 (2015).

## Supplementary Material

Supplementary Information Supplementary Figures 1-10
