# Supplementary material for: Restoration of skilled locomotion by sprouting corticospinal axons induced by co-deletion of PTEN and SOCS3
Source: Nat Commun. 2015 Nov 24;6:8074. doi: 10.1038/ncomms9074 (PMC4662086; doi:10.1038/ncomms9074)
Supplement: Supplementary Information — Supplementary Figures 1-10 [file ncomms9074-s1.pdf]

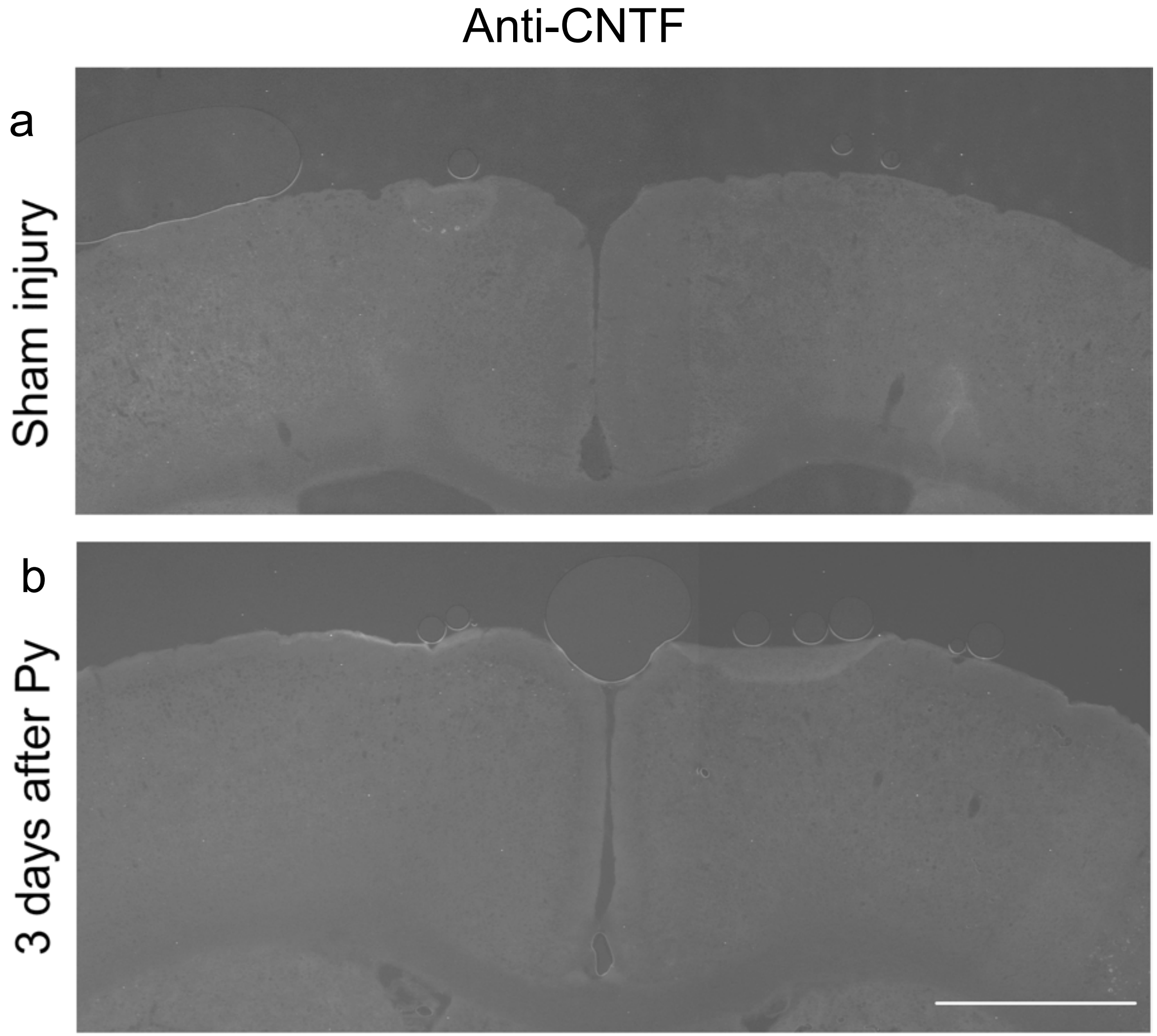

Supplementary Fig. 1

Supplementary Figure 1. CNTF is not up-regulated in the cortex after unilateral pyramidotomy. (a, b) Representative images of the cortical sections from the adult mice with a sham injury **(a)** or 3 days after a unilateral pyramidotomy **(b)** stained with anti-CNTF. Scale bar: 1000 μm.

Rosa-Lox-Stop-Lox-Tomato mouse cortex

Layer V

a

AAV-CreERT2
No Tamoxifen

b

AAV-CreERT2
Tamoxifen

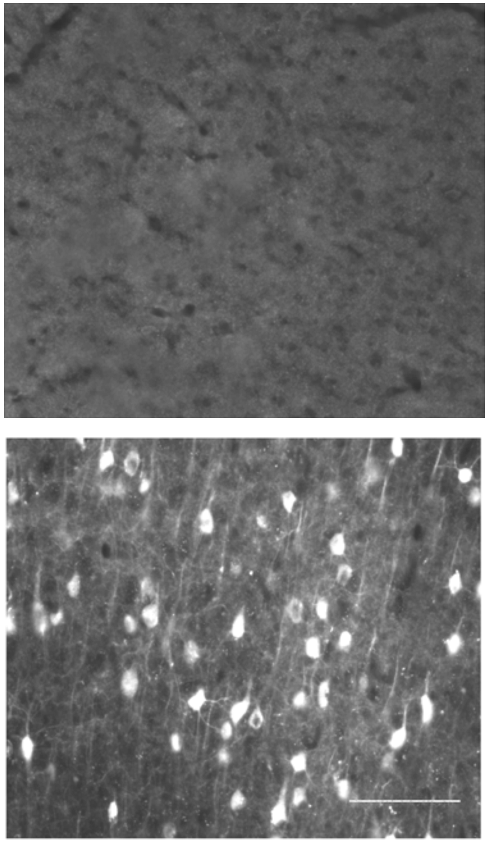

Supplementary Fig. 2

Supplementary Figure 2. AAV-CreERT2-mediated reporter Tomato expression in cortical neurons in the adult mice upon tamoxifen induction. Images from the cortical section from the reporter mice (Rosa-lox-stop-lox-Tomato) which received the cortical injection of AAV1-CreERT2 at the neonatal age and with **(b)** or without **(a)** tamoxifen induction at 8 weeks. With tamoxifen induction, many cortical neurons, including the pyramidal neurons in the layer V, express the Tomato reporter. Scale bar: 100 μm.

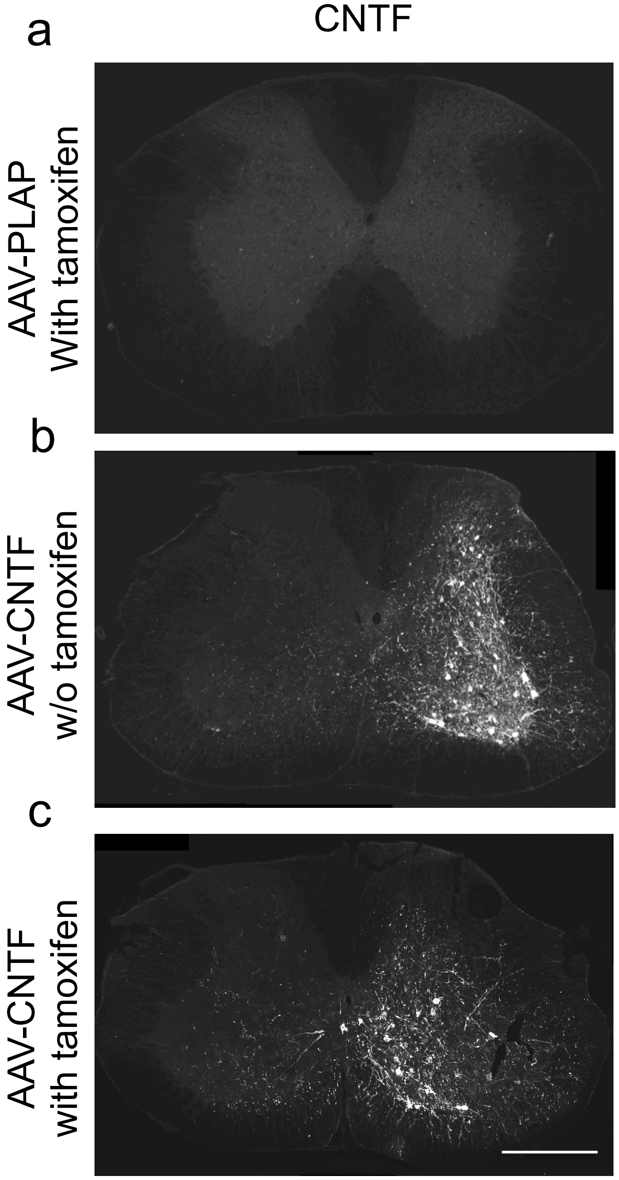

Supplementary Fig. 3

Supplementary Figure 3. CNTF expression in the cervical spinal cord after unilateral AAV-PLAP or AAV-CNTF intraspinal injection. (a-c) CNTF immunostaining of cervical 7 spinal cord transverse sections from SOCS3 $^{f/f}$ mice with neonatal cortical injection of AAV1-CreERT2 and tamoxifen **(a, c)** or oil **(b)** i.p. injection at the age of 6 weeks, intraspinal injection of AAV2-PLAP **(a)** or AAV2-CNTF **(b,c)** in the adult (~20 weeks). Scale bar: 500 μm.

BDA labeled CST in dorsal column

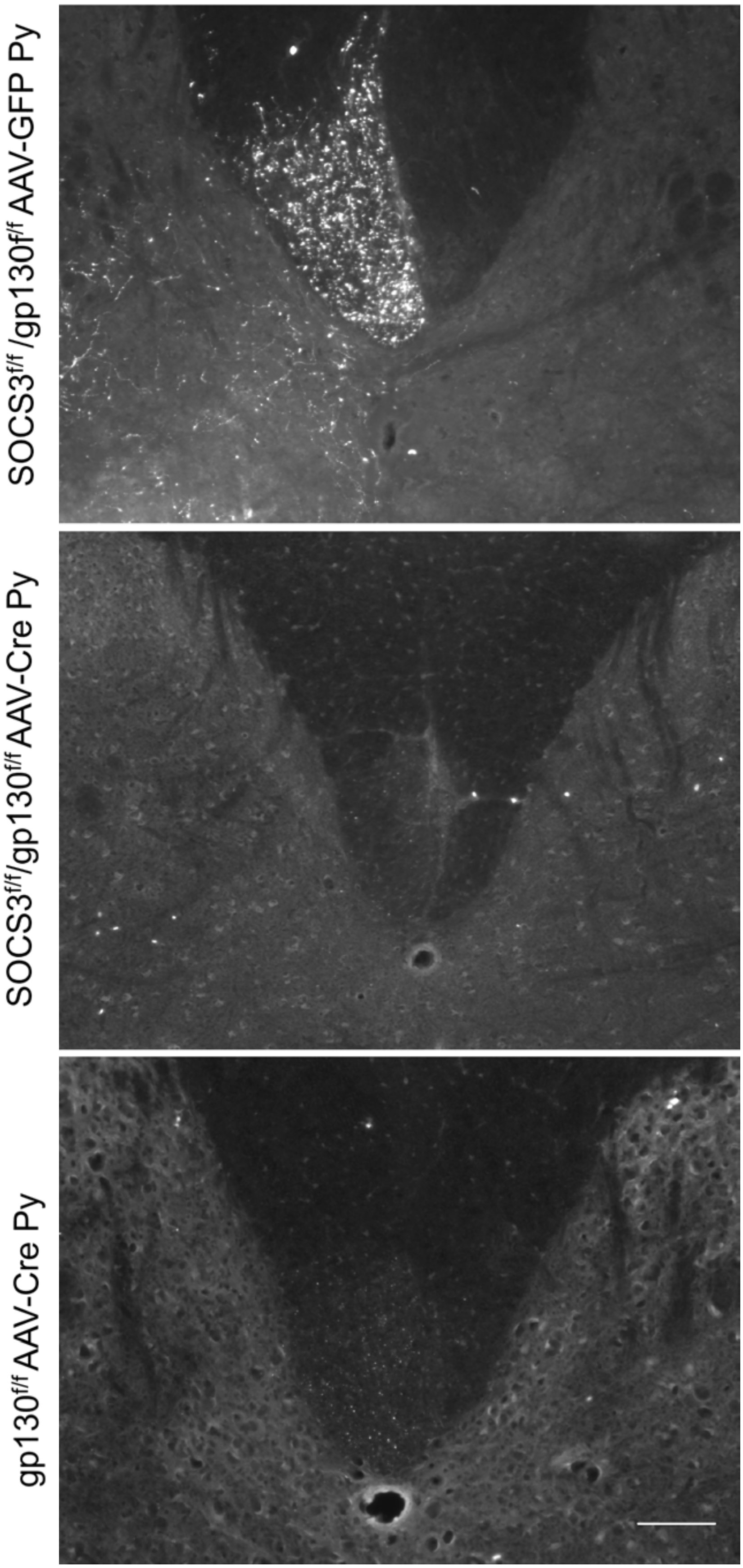

Supplementary Fig. 4

Supplementary Figure 4. Dramatically reduced BDA-labeled CST axons in gp130$^{f/f}$ or gp130$^{f/f}$/SOCS3$^{f/f}$ mice with neonatal AAV1-Cre cortical injection. Representative images of spinal cord sections from SOCS3$^{f/f}$/gp130$^{f/f}$ mice with cortical injection of AAV-GFP (upper panel) or AAV-Cre (middle panel), or gp130$^{f/f}$ mice injected with AAV-Cre (lower panel) at the neonatal age, unilateral pyramidotomy at the age of 4-6 weeks, and BDA tracer injection to the intact side of sensoromotor cortex in additional 4 week. The CST axons in the main dorsal column tract were labeled in the control, but not gp130 or gp130/SOCS3 deleted mice. Scale bar: 100 μm.

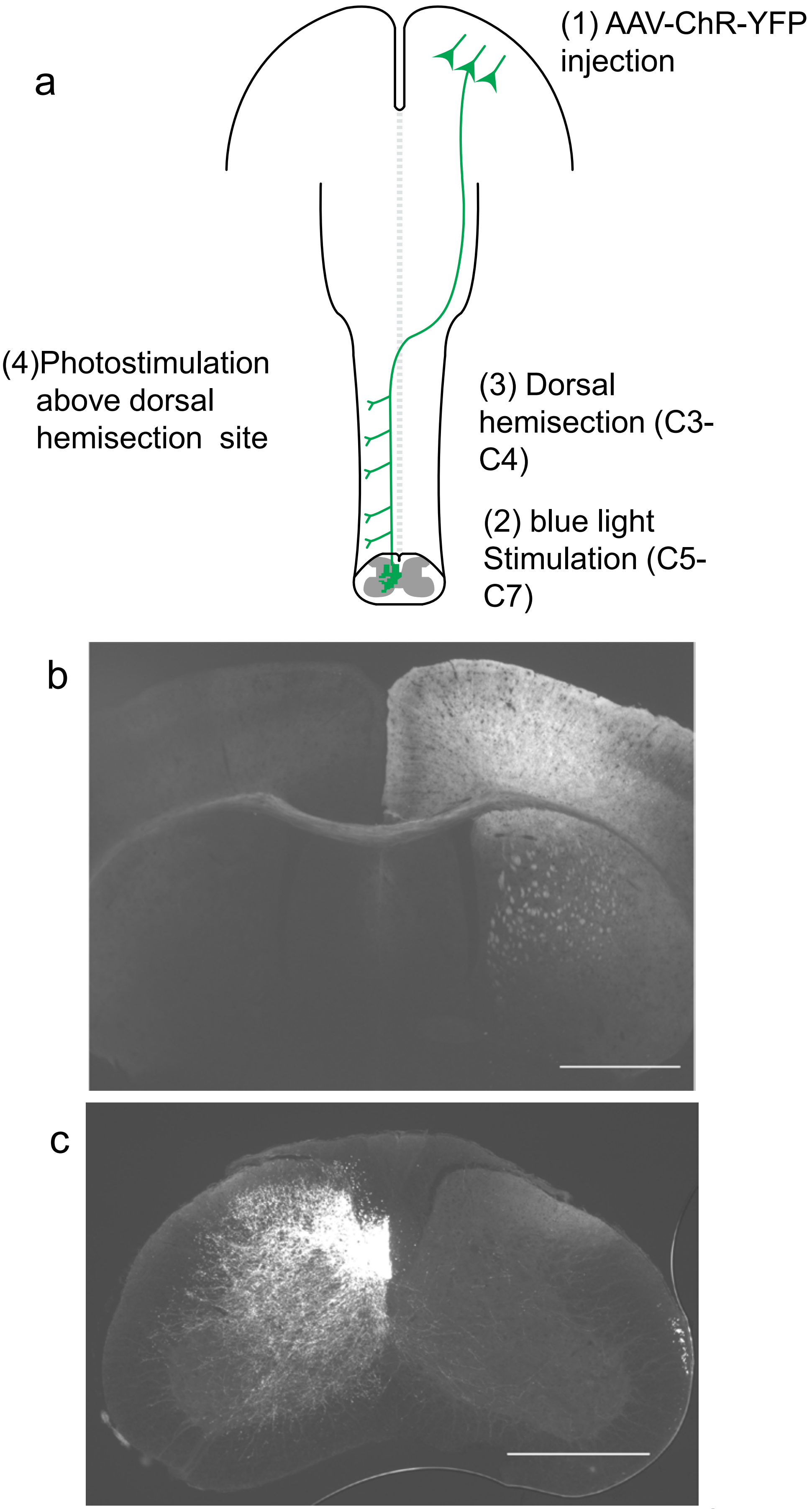

Supplementary Fig. 5

Supplementary Figure 5. Characterization of optogenetic stimulation of CST axons in the spinal cord. (a) Paradigm of optogenetic stimulation experiment. Mice first received a unilateral injection of AAV1-ChR-YFP on the cortex at P1 (1) and subjected to optogenetic stimulation at C5-C7 levels (2) at the age of 6-8 weeks. In some animals, optogenetic stimulation was placed above the lesion (4), after a C3 dorsal hemisection (3). **(b, c)** Representative images showing the unilateral expression of ChR-YFP in the cortex **(b)** and CST axons in the spinal cord **(c)** after unilateral AAV1-ChR-YFP cortical injection. Scale bars: 1000 μm **(b)** and 500 μm **(c)**.

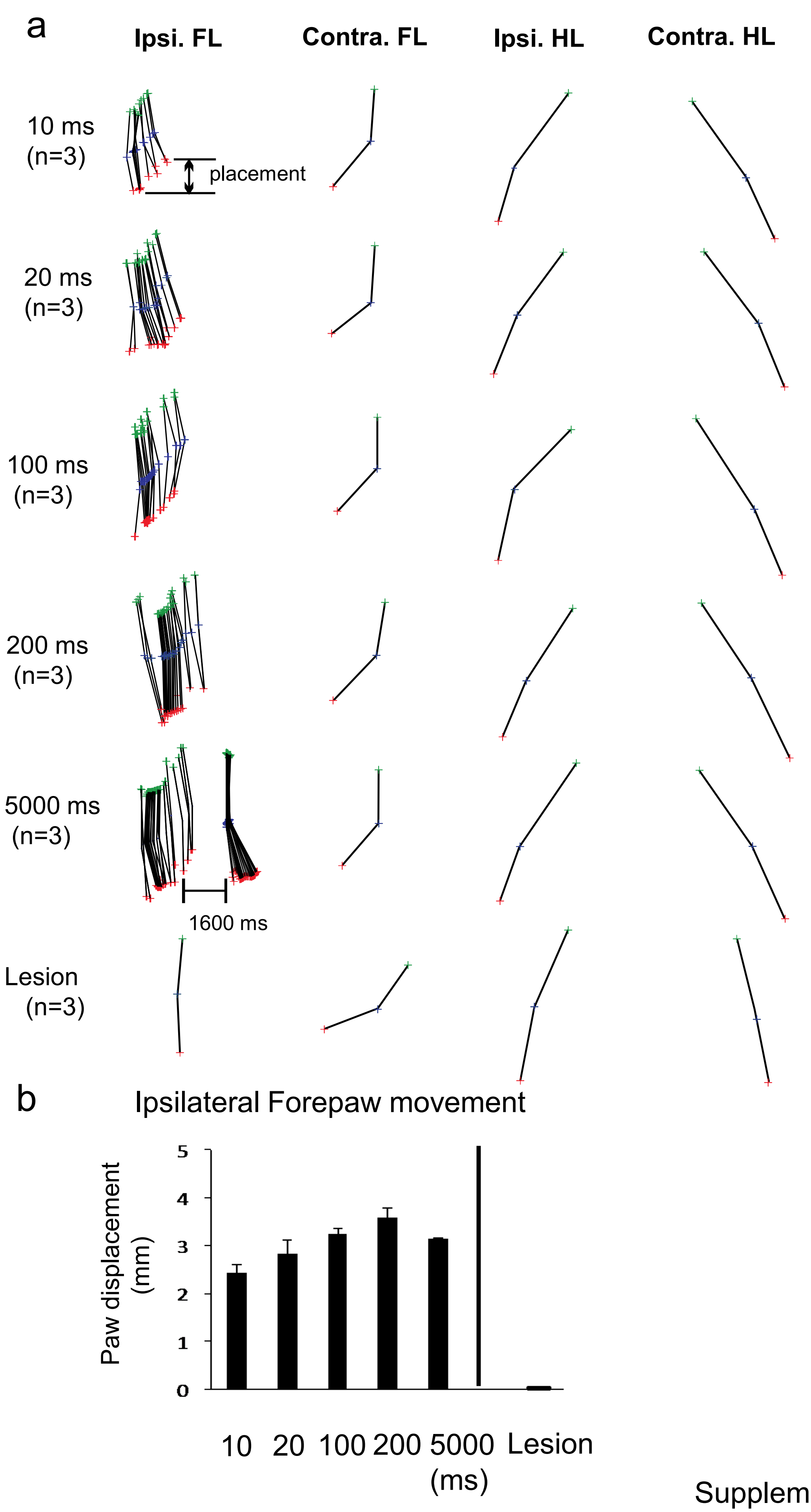

Supplementary Fig. 6

Supplementary Figure 6. Characterization of limb movement induced by photostimulation-mediated specific CST stimulation in intact animals.

(a) Representative paw movement trajectories in wild type animals with AAV1-ChR-YFP cortical injection induced by optogenetic stimulation with different durations at the C5-C7 levels. Note that only ipsilateral forelimb movement was elicited upon the stimulation. A C3 dorsal hemisection eliminates the limb movement induced by optogenetic stimulation at the spinal cord above the lesion (bottom panel). **(b)** Quantification of paw placement after optogenetic stimulations. Three animals were used for quantification and three trajectories were quantified per mouse.

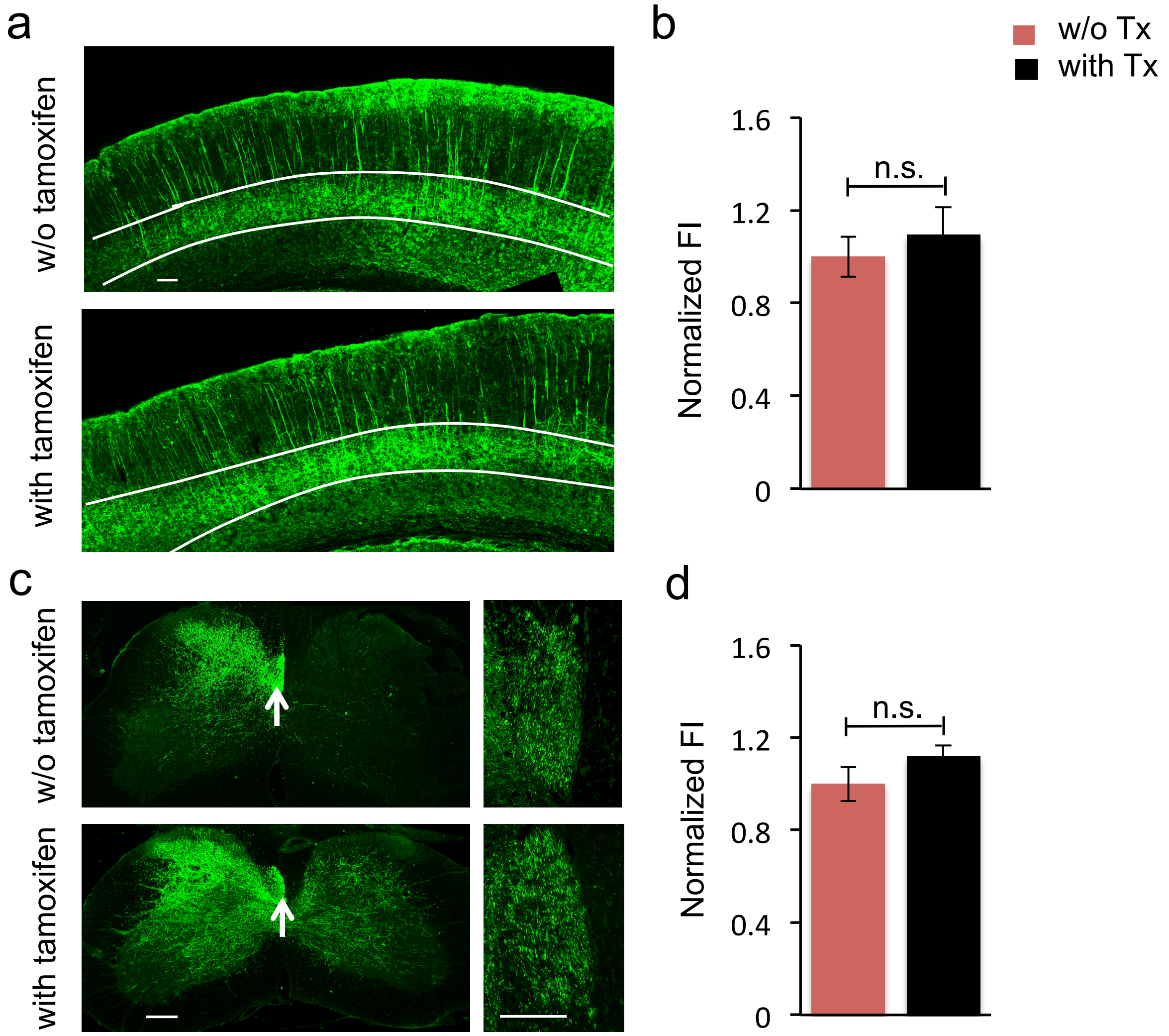

Supplementary Fig. 7

Supplementary Figure 7. Expression of channelrhodopsin-yellow fluorescent protein (ChR2-YFP) in the cortex and spinal cord in SOCS3/PTEN deleted and control mice. (a, c) Representative images of transverse sections of the cortex **(a)** and the spinal cord **(c, left column)** in SOCS3$^{f/f}$/PTEN$^{f/f}$ animals without (upper panel) or with (lower panel) tamoxifen induction. White lines in **(a)** delineated cortex layer V. Arrows in **(c)** pointed to the dorsal funiculus, where the main CST tracts travel in the spinal cord. Zoomed in images of the dorsal funiculus were showed in **(c, right column)**. Scale bars: 100 μm. **(b,d)** Comparison of the averaged fluorescent intensity of the cortex layer V **(b)** and spinal cord dorsal funiculus **(d)** between SOCS3$^{f/f}$/PTEN$^{f/f}$ animals with or without tamoxifen induction. n.s. no statistical significance. Student's *t* test (n=3,3).

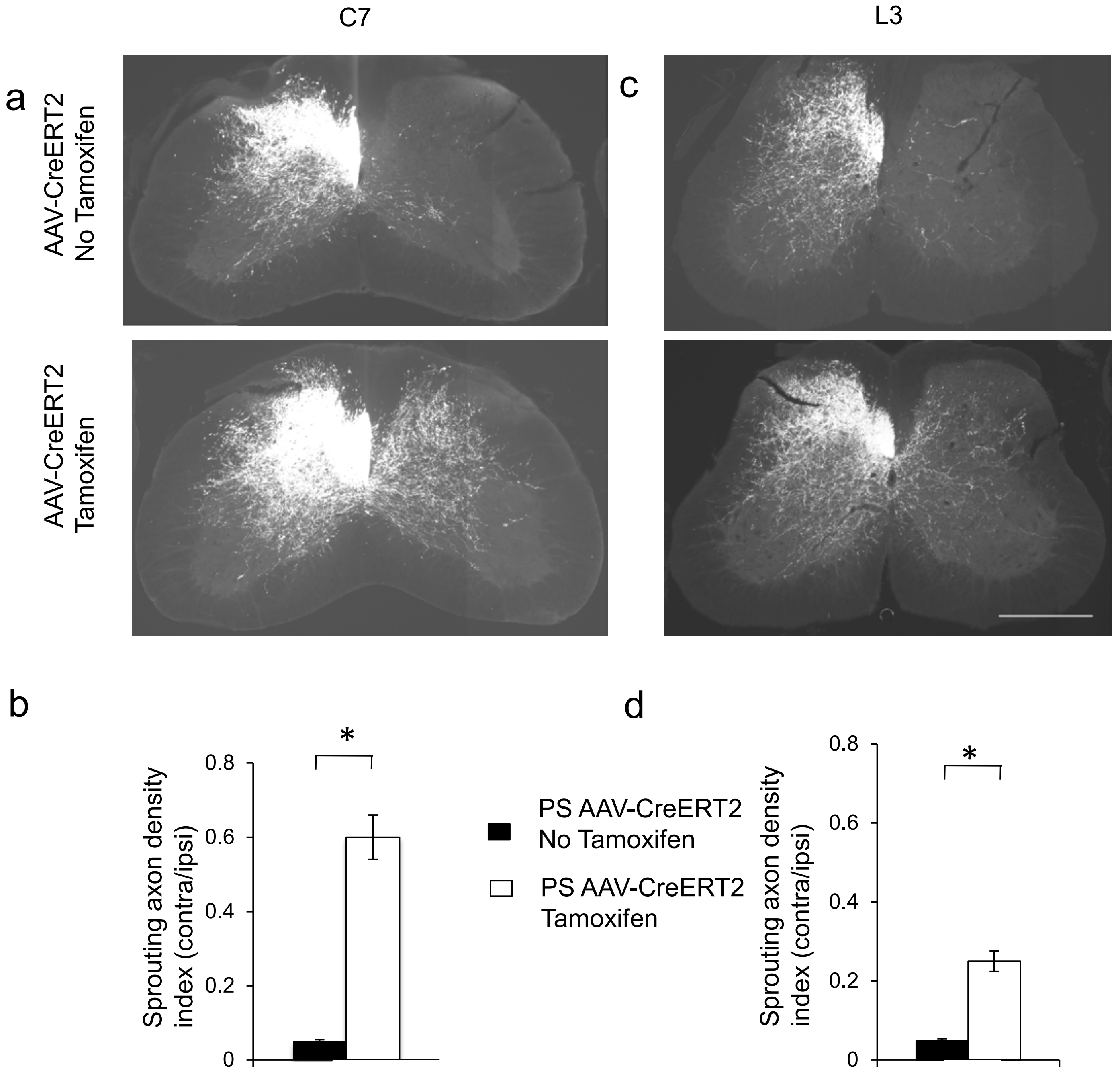

Supplementary Fig. 8

Supplementary Figure 8. CST sprouting at both cervical and lumbar levels after PTEN/SOCS3 deletion in adult cortical neurons. (a, c) Representative images of the spinal cord sections at C7 **(a)** or L3 **(c)** level from $PTEN^{f/f}/SOCS3^{f/f}$ mice with neonatal AAV1-CreERT2 and AAV-ChR-YFP injection, with or without tamoxifen induction at the age of 6 weeks, unilateral pyramidotomy at the age of 8 weeks. The CST axons at these spinal cord sections were indicated by the YFP signal (without immunostaining). **(b, d)** Quantification of the sprouting axon density at the cervical **(b)** or lumbar **(d)** levels. *t* test, $p < 0.01$.

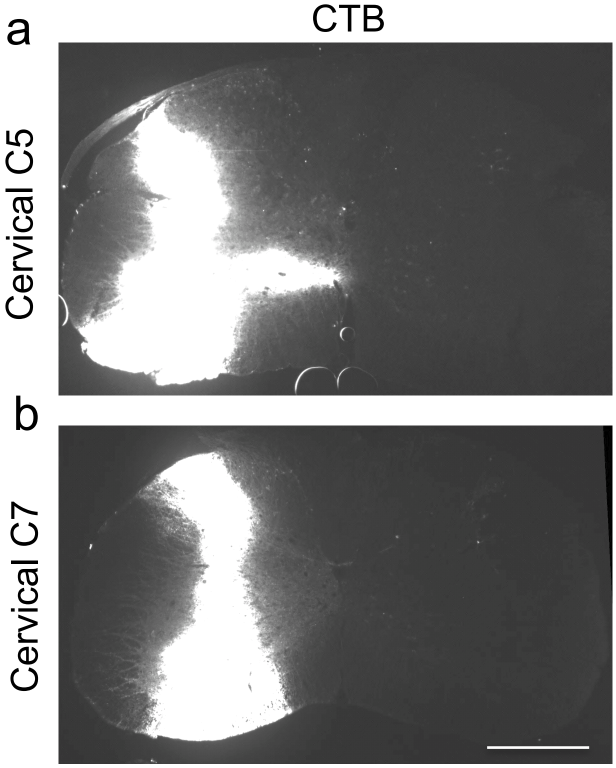

Supplementary Fig. 9

Supplementary Figure 9. Unilateral intraspinal injection to the cervical spinal cord C5-C7. (a,b) Cholera toxin B subunit (CTb) conjugated with Alexa Fluor® 555 was co-injected with HiRet-FLEX-DTR virus to the denervated side into the cervical spinal cord from C5 **(a)** to C7 **(b)**. Notice that Ctb was well circumscribed in the denervated side. Scale bar: 500 μm.

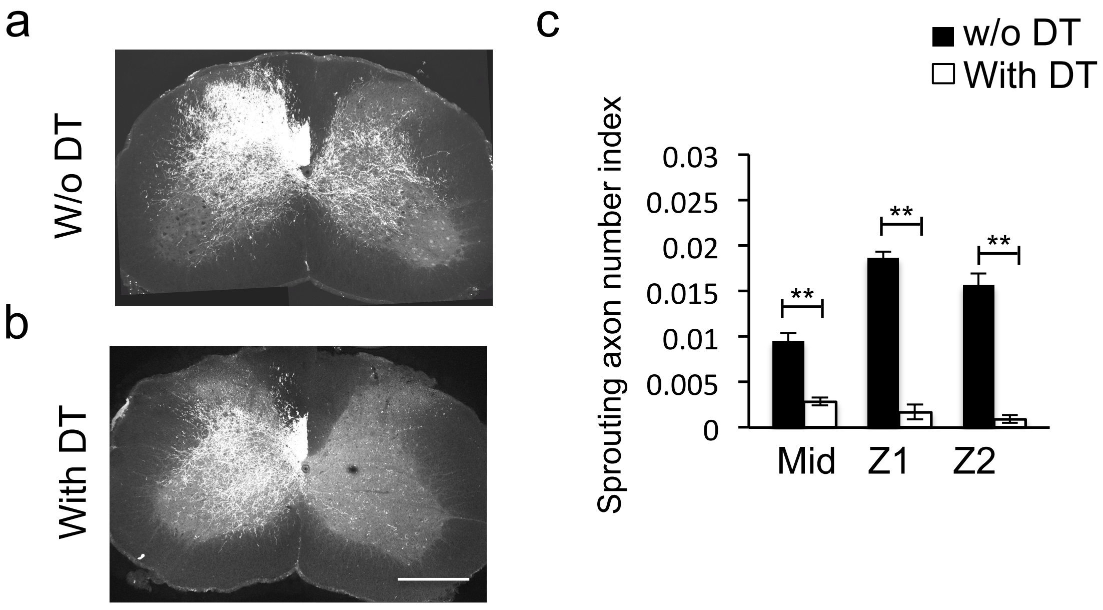

Supplementary Fig. 10

Supplementary Figure 10. Significant reduction of the collaterally sprouted axons in the denervated spinal cord after diphtheria toxin (DT) administration. (a,b) Representative cervical 7 (C7) spinal cord images of PTEN$^{f/f}$/SOCS3$^{f/f}$ mice received cortical injection of AAV-CreERT2 at neonatal stage, tamoxifen at the age of 6 weeks, unilateral pyramidotomy at 10 weeks, lenti HiRet-FLEX-DTR at 20 weeks, without **(a)** or with **(b)** DT administration at 22 weeks. Scale bar: 500 μm. **(c)** Quantification of crossing axons counted in different regions of spinal cord normalized against the numbers of labelled CST axons. **, $p < 0.01$, Student's *t* test. Three mice and three sections at C6-C7 per mouse were used in each group.
